# Behçet’s Disease as a Cause of Recurrent Deep Vein Thrombosis

**DOI:** 10.7759/cureus.85585

**Published:** 2025-06-09

**Authors:** Aazaz Rehman, William Monks, Rawya Ahmed, Nigel Marchbank

**Affiliations:** 1 Respiratory Medicine, University Hospitals Sussex NHS Foundation Trust, Brighton, GBR; 2 Radiology, University Hospitals Sussex NHS Foundation Trust, Brighton, GBR

**Keywords:** arterial aneurysm, atypical chest pain, behcet’s syndrome, behçet’s syndrome, complications of anticoagulation, deep vein thrombosis (dvt)

## Abstract

Behçet’s disease (BD) is a rare multisystem vasculitis that can present with various clinical manifestations, including recurrent venous thromboembolism. This case report details the diagnostic journey of a man in his early 40s who initially presented with deep vein thrombosis (DVT) but subsequently developed multiple thrombotic events despite appropriate anticoagulation therapy. His illness trajectory highlights the importance of a detailed clinical history and a comprehensive diagnostic approach, particularly when symptoms evolve over time and involve multiple organ systems.

DVT is a common condition that can arise due to immobility, malignancy, or thrombophilias. However, in patients with recurrent DVTs or unexplained progression despite treatment, clinicians must consider alternative systemic causes such as autoimmune disorders. This case highlights the diagnostic challenges encountered when BD presents with vascular symptoms, resulting in delayed recognition of the underlying inflammatory pathology.

In this report, we discuss the critical role of thorough history-taking, the importance of considering BD as a differential in unexplained thrombotic events, and the potential consequences of delayed diagnosis. The findings highlight how BD can mimic other conditions, emphasizing the importance of maintaining a broad differential when evaluating patients with recurrent thromboembolism. Through a review of the patient’s case and relevant literature, we highlight the necessity of prompt recognition and early initiation of immunosuppressive therapy to prevent life-threatening complications such as pulmonary artery aneurysms.

This case reinforces the value of a holistic diagnostic approach in young patients presenting with recurrent DVTs. It illustrates the need for vigilance in recognizing uncommon causes of thrombosis and underscores the importance of collaborative care among rheumatology, hematology, and vascular specialists in managing such patients.

## Introduction

Deep vein thrombosis (DVT) is a common yet potentially life-threatening condition encountered in clinical practice worldwide. It presents with clinical symptoms in 1-2 individuals per 1000 each year, although it can often be asymptomatic [1]. While known risk factors such as immobility, recent surgery, malignancy, and inherited thrombophilias contribute significantly to DVT development, some cases fall outside these typical categories and require a more nuanced diagnostic approach [1]. The mainstay of treatment is anticoagulation, most commonly with direct oral anticoagulants (DOACs). If left untreated, DVT can lead to serious complications, including fatal pulmonary embolism.

In complex presentations where recurrent DVTs occur despite appropriate anticoagulation, clinicians must broaden their differential diagnoses beyond conventional risk factors. Thorough history-taking and a holistic evaluation of multisystem involvement play a pivotal role in identifying underlying conditions that might predispose individuals to thrombosis. This case report illustrates how Behçet’s disease (BD), a rare multisystem vasculitis, can be an overlooked cause of recurrent DVTs.

We describe the diagnostic course of a previously healthy man in his 40s who initially presented with a single DVT but subsequently developed multiple thrombotic episodes. This case highlights the importance of maintaining a high index of suspicion for inflammatory and autoimmune etiologies in atypical or recurrent thrombotic events.

## Case presentation

A man in his 40s presented to his general practitioner (GP) with acute right-sided calf swelling. Two weeks prior, he had experienced high fevers, neck pain, headache, and anorexia, accompanied by transient right-sided pleuritic chest pain and breathlessness. The illness was severe enough to confine him to bed for 10 days. Four days before the current presentation, he presented to a neighboring emergency department (ED) and was discharged with antibiotics, although no definitive diagnosis was made. Despite adherence to the prescribed antibiotics, he remained unwell, reporting persistent high fevers and sweating. At presentation, his GP suspected an acute right DVT, with recent immobility and dehydration considered potential provoking factors. It was noted that a chest X-ray performed four days earlier was normal, although his C-reactive protein was markedly elevated. The patient requested referral to a different hospital and was subsequently seen at our institution’s ED, where repeat blood tests were performed (Table 1), and treatment with low-molecular-weight heparin (LMWH) was initiated.

**Table 1:** Summary of blood test results from initial and second emergency department presentations

Test	First presentation result	Second presentation result	Range
Hemoglobin	143	139	135-145 g/L
White blood cell count	13.6	11.8	3-8 x 10⁹/L
Neutrophils	11.7	9.5	1-6 x 10⁹/L
Lymphocytes	0.9	1.2	1-3 x 10⁹/L
C-reactive protein	273	302	0-5 mg/L
Erythrocyte sedimentation rate	107	111	0-14 mm/hour
D-dimer	19		0-0.5 mcg/mL

Two days after the initial presentation, the patient was reviewed in the ambulatory assessment unit, where Doppler ultrasound confirmed a right calf DVT extending into the popliteal vein.

He had no significant past medical history and was not on any regular medications. He was previously fit and well, with no history of smoking and occupational risk factors. A comprehensive respiratory viral panel, including a COVID-19 PCR test, was negative. He reported some symptomatic improvement, and a repeat chest X-ray remained normal.

No further investigations were undertaken aside from repeat blood tests (Table 1). Despite ongoing antibiotic therapy for an undefined illness, no objective clinical findings explained the persistently elevated inflammatory markers. He was advised to complete the course of antibiotics, return for review in one week, and was commenced on a direct oral anticoagulant (DOAC).

One week later, the patient presented again for a follow-up, reporting worsening leg pain. A repeat Doppler ultrasound of the right leg confirmed the proximal extension of the thrombus into the mid-femoral vein. As a result, he was switched back to LMWH. He was advised to return for further review in one week. At the subsequent review, he reported improvement in leg pain and no new symptoms. In light of the thrombus extension, a vascular consultation was sought, and he was transitioned from LMWH to warfarin.

**Figure 1:**
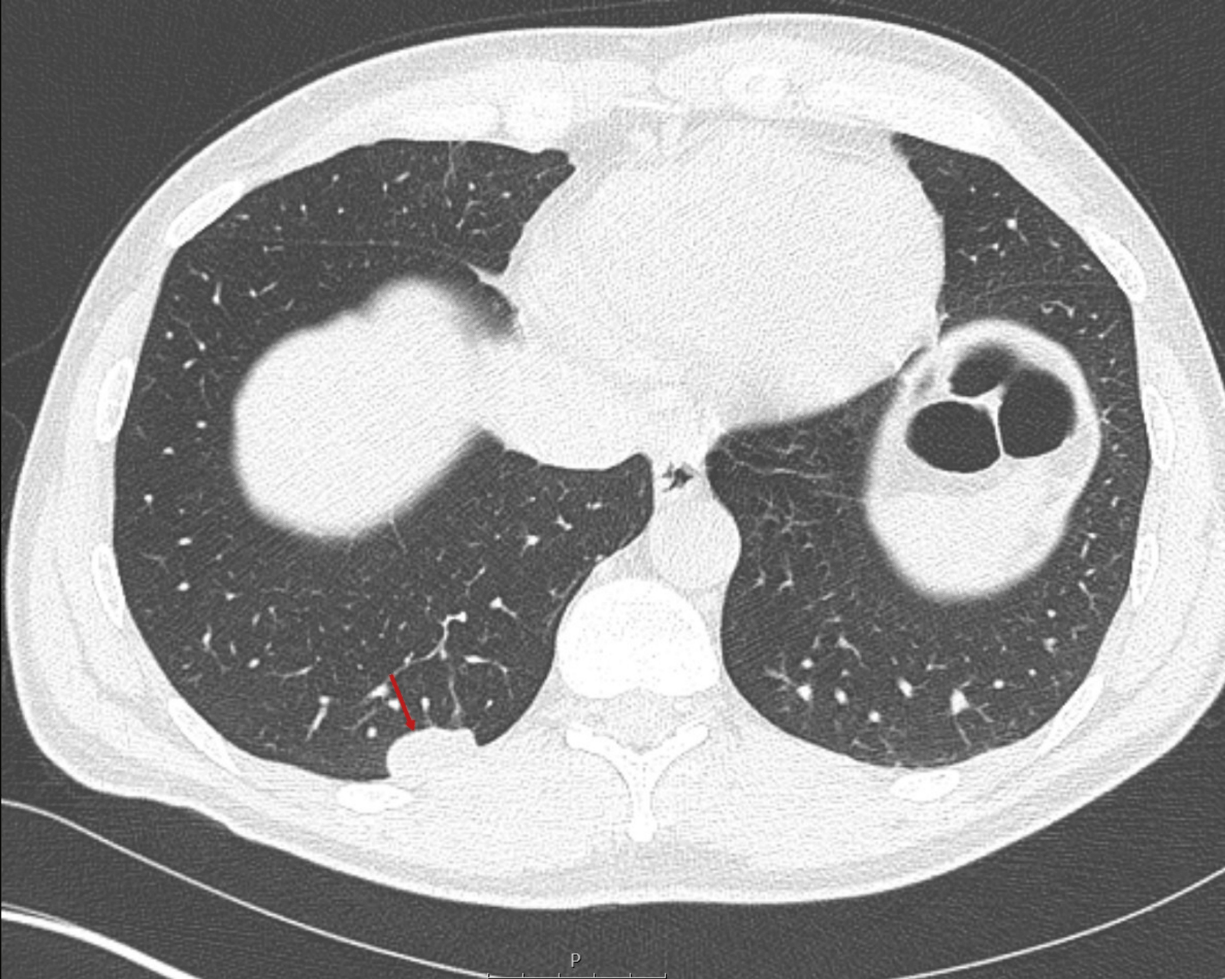
Coronal CT image showing a large flattened nodule in the right lower lobe

A CT scan of the abdomen and pelvis was performed to evaluate for occult malignancy, which was negative. However, the scan incidentally revealed multiple subpleural nodules in the right lower lobe (Figure 1). A non-contrast chest CT was initially considered for routine nodule surveillance; however, due to the unusual flattened morphology of the nodules, the differential diagnosis was broadened to include pulmonary artery aneurysms. As a result, a CT pulmonary angiogram (CTPA) was requested. He was reviewed again one week later and reported continued symptom improvement. He was subsequently discharged to primary care with safety-netting advice and follow-up arranged in the venous thromboembolism (VTE) clinic.

At this stage, several factors remained concerning. Pertinent in his presentation was a febrile illness without definitive signs or symptoms of infection. Inflammatory markers remained elevated more than two weeks into his illness, an abnormality unlikely to be explained solely by the presence of a DVT or a self-limiting viral infection. The persistence of fever despite antibiotic therapy should have prompted consideration of non-infectious inflammatory or autoimmune causes. Although documentation suggested minimal systemic upset, the documented progression of DVT despite anticoagulation is a clinically significant event. This should have triggered a more comprehensive history, targeted physical examination, and consideration for inpatient management to ensure optimal anticoagulation and diagnostic workup. Within the framework of Virchow’s triad, venous stasis is the least likely mechanism. Instead, a hypercoagulable state or endothelial dysfunction are more plausible contributors and carry the potential for serious complications.

Five weeks following his initial presentation to his GP, the patient developed left calf pain during exercise, accompanied by breathlessness and palpitations. A Doppler ultrasound confirmed a left-sided DVT, prompting referral for a hematology review due to recurrent thrombotic events despite ongoing anticoagulation therapy.

**Figure 2:**
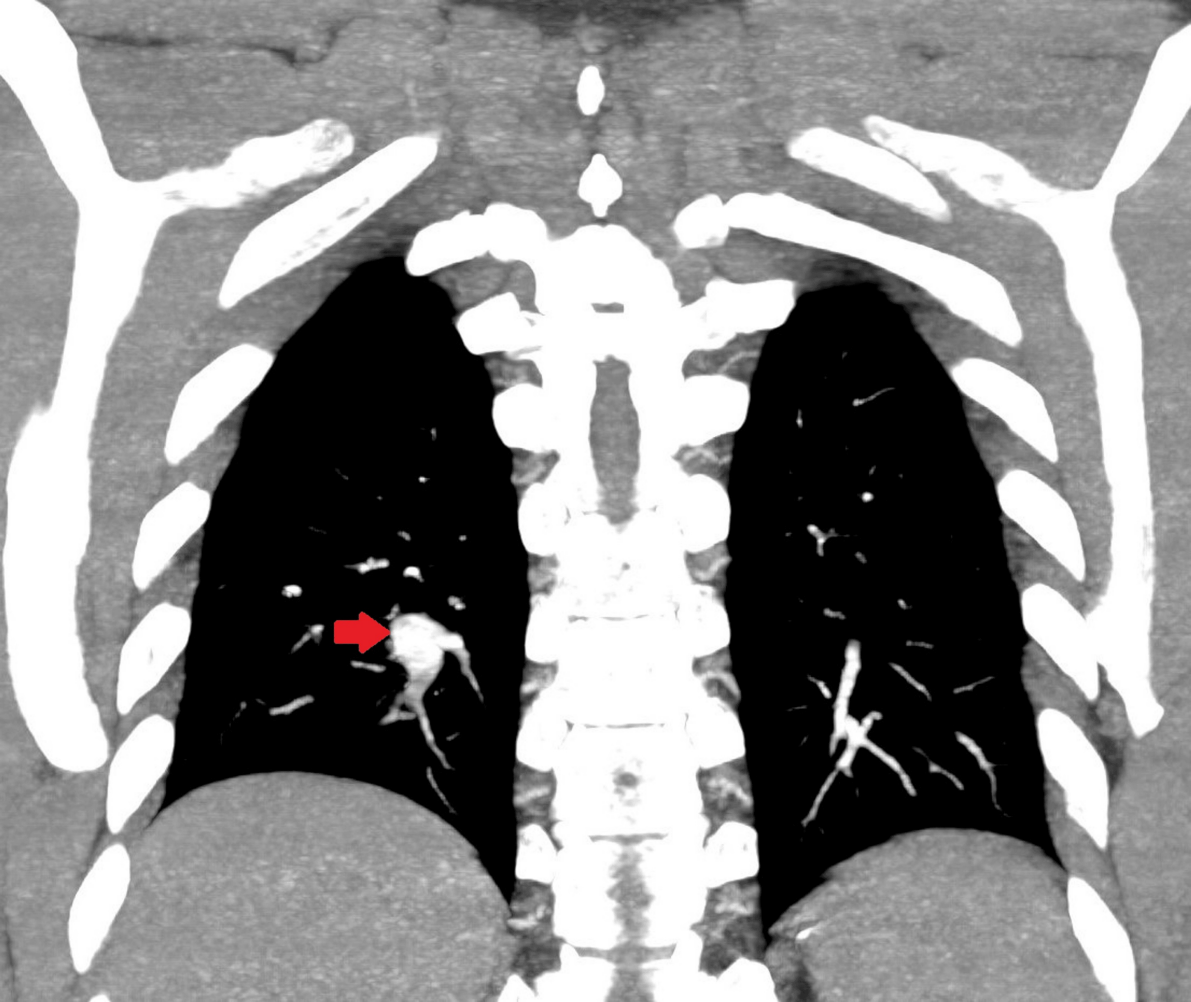
CT pulmonary angiogram (coronal view) demonstrating aneurysmal dilatation of the posterior segmental branch of the right lower lobe pulmonary artery

The peripheral distribution of the previously noted pulmonary nodules, in the context of his clinical presentation, raised the suspicion of segmental pulmonary embolism with distal infarction. A CTPA performed six weeks later revealed aneurysmal dilatation of the posterior segmental branch of the right lower lobe pulmonary artery with a small peripheral thrombus (Figure 2). The subpleural lesions identified on earlier imaging had resolved and were presumed to represent small pulmonary infarcts distal to the aneurysm with arterial thrombosis. The patient was subsequently reviewed in the chest clinic three months after his initial presentation, where the follow-up chest CT findings were discussed.

During this consultation in the respiratory nodule clinic, a detailed history was taken. The patient reported having bouts of oral ulcers occurring approximately every two months, for as long as he could remember, for which he had self-managed with over-the-counter topical agents. He denied any history of genital ulcers. He described a lifelong tendency for minor skin injuries to heal slowly, often forming pustules that he would manually express to facilitate resolution. He reported no gastrointestinal symptoms but noted bilateral ankle pain that had gradually worsened over the past year. Additionally, he experienced recurrent headaches. Although he had no current ocular symptoms, he had a history of right-sided retinal detachment.

The patient was of Caucasian descent with no Mediterranean ancestry. His family history was notable for a parent who died from lung cancer and a brother who suffered sudden cardiac death in his 30s. There was no known family history of VTE. A working diagnosis of BD was considered, and the patient was referred to the rheumatology team for further assessment and diagnosis. Subsequent assessment excluded other systemic vasculitides, and testing for antiphospholipid syndrome was negative.

Investigations

Differential Diagnosis

In patients presenting with recurrent DVTs or extension of recently diagnosed VTE, exclusion of malignancy is a priority. However, in younger individuals, inherited thrombophilias must also be considered. While the overall management of VTE in the context of hereditary thrombophilia remains similar to other causes, genetic testing is typically deferred until anticoagulation can be safely paused. The most common inherited thrombophilia is the Factor V Leiden mutation, which leads to resistance to activated protein C. Notably, this resistance may also be acquired, particularly in systemic lupus erythematosus (SLE) and antiphospholipid syndrome (APS) [2]. These autoimmune conditions often present with multisystem involvement, including mucocutaneous lesions and joint symptoms.

In cases of VTE extension in a young man, BD should be a key differential diagnosis. A thorough clinical history is critical to identify hallmark features, as BD remains a diagnosis of exclusion. Buerger’s disease is strongly associated with tobacco use, a factor not present in this patient. In contrast to connective tissue disorders, systemic vasculitides infrequently manifest with thrombotic events.

Respiratory symptoms often reflect diffuse lung parenchymal involvement, although discrete multiple lung nodules may be present. Incidental pulmonary nodules are increasingly identified through non-targeted imaging. In younger patients, these nodules are most often benign, inflammatory, or transient in nature, such as perifissural or intrapulmonary lymph nodes. However, multiple solid nodules with lower lobe distribution can raise suspicion for metastatic disease. While benign hamartomas also tend to be located basally, they are usually solitary. Other vascular malformations such as arteriovenous malformations require a contrast-enhanced CT for accurate characterization.

Treatment

Azathioprine 50 mg twice daily was initiated five months after his initial presentation. After a discussion with the hematology team, he was reverted to his original DOAC.

Outcome and Follow-Up

He was reviewed in the regional BD center 10 months after his initial presentation. At this time, his shortness of breath had improved, with fatigue being his predominant complaint. Clinical examination at this time revealed a single 2 mm intraoral ulcer on his left lateral tongue. Ophthalmologic assessment confirmed normal visual acuity and no signs of active inflammation. Blood tests revealed iron deficiency, and oral iron supplementation was initiated. Oral mouthwash containing betamethasone, doxycycline, and nystatin was prescribed to help with his ulcers. He had undergone human leukocyte antigen (HLA) testing at this point, which was negative for HLA-B51. However, the clinical features, such as oral ulcers, signs of pathergy, and pulmonary aneurysm, supported a clinical diagnosis of BD. The presence of a pulmonary artery aneurysm in the context of anticoagulation was considered for intravascular intervention but deemed unnecessary given that he was already established on targeted therapy and had had no hemoptysis at any point during his presentation. Regular interval chest CT scan follow-up is planned.

## Discussion

BD was described in 1937 as the presence of hypopyon, iritis, and orogenital ulcers [3]. A subsequent case series by Hughes and Stovin in 1959 described findings of DVT and pulmonary artery aneurysms called Hughes-Stovin syndrome (HSS) [4]. Over time, the consensus has become that HSS is a subset of BD and their treatment is the same [5]. Mouth and genital ulcers with inflamed cartilage (MAGIC) describe the hallmark features of BD, observed most commonly in regions across Asia, the Middle East, and Turkey [6]. Diagnosis of BD can be made using the international criteria for BD, which utilizes a points-based system [7]. Although HLA-B51 testing is not part of the criteria, it is commonly used to aid diagnosis and can affect the clinical phenotype of the disease [8].

The rate of vascular involvement in BD is somewhere between 14% and 16%, and in only 2% of presentations, a vascular lesion is the initial symptom [9,10]. Vascular BD has three subtypes: venous occlusions, arterial occlusions, and arterial aneurysms. The rate of vascular involvement in BD varies depending on different countries. In Turkey, vascular involvement is seen in up to 14% of patients, whereas in China, vascular involvement is seen in 17.98% of patients [9,11,12]. Of arterial involvements, pulmonary artery involvement is the most frequent [12]. This can occur as either aneurysms or thrombosis; our patient presented with both [12]. The prevalence of pulmonary artery involvement in BD patients is between 1.1% and 1.9% [13,14]. The majority of these patients are male, with most getting a diagnosis before the age of 30 [11,15]. The overall mortality of BD is between 5% and 8%, but rare, ruptured pulmonary artery aneurysms (PAAs) represent a major cause of mortality [16]. Mortality rates of up to 50% within 36 months have been associated with PAAs secondary to BD [8]. Early diagnosis and immunosuppressant treatment can drastically improve this, with a follow-up study showing a mortality rate of 13% in patients who had received prompt treatment [9]. It is, therefore, important to keep such diagnoses in mind when faced with incalcitrant DVTs. Vessel inflammation in BD can cause both thrombosis and aneurysmal dilatation. Steroids and immunosuppressive agents such as azathioprine and cyclophosphamide are the mainstay of treatment [17]. 

It is important to note in this case that the patient presented with vague symptoms and an elevated CRP, which was misdiagnosed. Only when the patient was seen in the respiratory nodule clinic that a thorough history was obtained. A history of recurrent oral ulcers and slow-healing wounds was not elucidated on acute presentations despite their atypical nature. A careful history could have led to quicker diagnosis and prompt immunosuppressant treatment.

Low-risk pulmonary emboli and DVTs are managed in ambulatory care. Follow-up is organized in 3-6 months in a VTE clinic depending on etiology [18]. Recurrent DVTs despite anticoagulation require further prompt investigation. The first thought must be adherence to treatment. If adherence is not an issue, then malignancy must be ruled out with a CT chest, abdomen, and pelvis to look for solid tumors. One study shows a 36% rate of malignancy in patients with recurrent DVTs on anticoagulation [19]. Thrombophilias must also be screened for, though one study shows only a 0.9% recurrence rate in patients on anticoagulation [20]. If the patient has been treated with LMWH then heparin-induced thrombocytopenia should be ruled out.

## Conclusions

This case underscores the critical importance of thorough and systematic history-taking, particularly when patients present with atypical or unexplained features. Early recognition and diagnosis of rare underlying conditions are vital, as timely treatment can significantly reduce morbidity and mortality. The identification and interpretation of incidental lung nodules should ideally occur in specialist clinics, where multidisciplinary input, particularly from radiology and respiratory teams, can help distinguish between benign and more serious pathologies. Recurrent DVTs, especially those occurring despite appropriate anticoagulation, should prompt comprehensive investigation to exclude malignancy.
